# Intestinal Helminth Infections and Their Association with QuantiFERON-TB Gold Plus Test Performance in an Endemic Setting, Northwest Ethiopia

**DOI:** 10.2147/IDR.S476492

**Published:** 2024-10-17

**Authors:** Yohannes Zenebe, Markos Abebe, Abaineh Munshea, Gizachew Yismaw, Meaza Zewde, Mekdelawit Alemayehu, Roland Lang, Meseret Habtamu

**Affiliations:** 1 Department of Medical Laboratory Science, College of Medicine and Health Sciences, Bahir Dar University, Bahir Dar, Ethiopia; 2 Health Biotechnology Division, Institute of Biotechnology, Bahir Dar University, Bahir Dar, Ethiopia; 3 Mycobacteria and Other Bacterial Diseases Research Division, Armauer Hansen Research Institute, Addis Ababa, Ethiopia; 4 Department of Biology, Science College, Bahir Dar University, Bahir Dar, Ethiopia; 5 Department of Microbiology, Amhara Public Health Institute, Bahir Dar, Ethiopia; 6 Institute for Clinical Microbiology, Immunology and Hygiene, University Hospital of Erlangen, Friedrich-Alexander Universität Erlangen-Nürnberg, Erlangen, Germany

**Keywords:** intestinal helminths, LTBI, QFT-Plus, *MTB*, Ethiopia

## Abstract

**Background:**

Timely detection and treatment of latent TB infection (LTBI) is part of WHO’s strategy against tuberculosis (TB). Helminth infections can modulate immune responses, potentially impacting the performance of interferon-gamma release assays (IGRAs) such as the QuantiFERON-TB Gold Plus (QFT-Plus). This study evaluated the association between helminth infections and QFT-Plus results among participants from a TB-endemic region.

**Methods:**

A cross-sectional study was conducted from October 2022 to March 2023 in Bahir Dar, Ethiopia. Stool samples of 314 potential participants were examined for helminths using wet mount and Kato-Katz techniques. LTBI was assessed by QFT-Plus from a total of 100 gender-matched helminth-positive and -negative participants. The association between helminth infection status, egg count, and QFT-Plus positivity was analyzed, and *p* values *<0.05* were considered significant.

**Results:**

Overall, 53 of 314 screened participants were infected with helminths (16.9%), with *A. lumbricoides* (47.2%) and hookworm (30.2%) as most prevalent species. The overall QFT-Plus positivity rate was 30.0%, with similar rates observed between helminth-positive and helminth-negative participants. Although QFT-Plus positivity was slightly lower in hookworm carriers (25%) compared to those with *A. lumbricoides* (32%), a higher-than-median hookworm egg burden was significantly associated with reduced QFT-Plus positivity (*P = 0.029*). QFT-Plus positivity was significantly higher among male participants than females (*P* = *0.032*).

**Conclusion:**

While overall helminth infection status did not significantly affect QFT-Plus positivity, higher hookworm burden was associated with reduced QFT-Plus reactivity. These findings suggest that the type of helminth and infection intensity, rather than its mere presence, may influence IGRA performance. Further studies with larger sample sizes are warranted to understand the species-specific effect of helminth infection on immune modulation of the host.

## Introduction

Tuberculosis (TB) remains a major public health threat worldwide.^1^ Exposure of a person with *Mycobacterium tuberculosis (M.tb)* can result in one of the three outcomes: the host’s immune system controls and clears the pathogen immediately; or *M.tb* may proliferate, causing primary TB disease; or the host’s immune system may contain the bacilli controlling proliferation, resulting in latent tuberculosis infection (LTBI). A person with LTBI does not have any symptoms but can potentially develop active TB disease later in life depending on the individual’s immune status.^2^

One-third of the world population is estimated to be infected with *M.tb* and more than 2 billion people are also infected with helminths globally.^3^ The two debilitating infectious agents co-exist, mainly in middle- and low-income countries,^3^ where Ethiopia is part of the region. Intestinal parasitic infection is the second leading cause of outpatient morbidity in Ethiopia with more than one-third of children infected with soil-transmitted helminth (STH) (4, 5).^4^,^5^ Studies found that *Ascaris (A). lumbricoides* is the most prevalent STH in Ethiopia, with one-third of the population being infected with this parasite.^6^,^7^ The study conducted from Arba-Minch, Ethiopia, revealed 26.3% co-infection of helminth and TB among adult participants, with the highest co-infection rate of *A. lumbricoides* followed by *hookworms* and *T. trichiura*.^8^

*M.tb* and helminth infection mount different host immune responses. Type 1 immune response with robust production of interferon-gamma (IFN-γ) is the primary immunity type required for controlling *M.tb* infection.^9^ IFN-γ is essential for macrophage activation, resulting in the stimulation of nitric oxide (NO) synthase and NO production, vitamin D-dependent autophagy, phagolysosome fusion, and bacterial cell death.^10–12^ In contrast, helminth infections are characterized by strong Type 2 immunity with higher anti-inflammatory response characterized by IL-4 and IL-13 production, which can impair the development of type-1 immunity and then dampen protective immune responses to *M.tb*.^13^ Therefore, helminth infection may promote the progression of LTBI to active TB disease and affect the diagnostic performance of IGRA tests.^14^,^15^ In reverse, a Th1-mediated immunity to *M.tb* infection may also favor an immune escape of helminths by down-regulation of the Th2 immune response.^16^

Early diagnosis and treatment of LTBI is a promising approach for the TB eradication program. Tuberculin skin test (TST) and IGRAs are the frequently used LTBI screening tools.^17^ The TST is commonly used and inexpensive but low in its specificity.^17^ Interferon gamma-release assays have higher specificity,^18^ and are recommended by the Centers for Disease Control and Prevention (CDC) and Infectious Disease Society of America (IDSA) as a method for latent tuberculosis screening.^19^,^20^ QFT-Plus is the latest generation LTBI screening IGRA test. The previous generations tested only IFN-γ production by the CD4 cells. However, the new QFT-Plus test now looks at not only the CD4 response (peptides from RD1-restricted protein antigens called ESAT-6 and CFP-10 in TB1) but also contains additional peptides (TB2) triggering *M.tb*-specific CD8 T cells.^21–23^

Concomitant intestinal helminth infection in patients with TB can skew the cytokine profile of the host toward a Th2 immune response, which may affect the screening performance of IGRA tests. Studies showed that purified protein derivative (PPD)-specific IFN-γ production and T-cell proliferation were improved with albendazole treatment of helminth-positive individuals.^24^,^25^ However, there is a lack of strong evidence for the impact of helminth infection on QFT-Plus test performance. Hence, the main objective of this study was to determine the effect of helminth infection on the performance of the QFT-Plus test for LTBI detection.

## Materials and Methods

### Study Population, Area and Design

An institution-based cross-sectional study was conducted from October 2022 to March 2023 at Shimbit Health Center, Bahir Dar city administration to determine the association between helminth infection and QFT-Plus test results. Bahir Dar is the capital city of Amhara National Regional State, located 565 km driving distance from the capital city of Ethiopia, Addis Ababa. Gender-matched study participants from outpatient setting were conveniently selected and screened for their helminth status with both wet mount and Kato-Katz methods at Shimbit Health Center. In addition, we identified a comparable number of individuals who tested negative for helminth infection, with no parasite eggs or larvae detected in their stool samples.

### Inclusion/Exclusion Criteria

**Figure 1:**
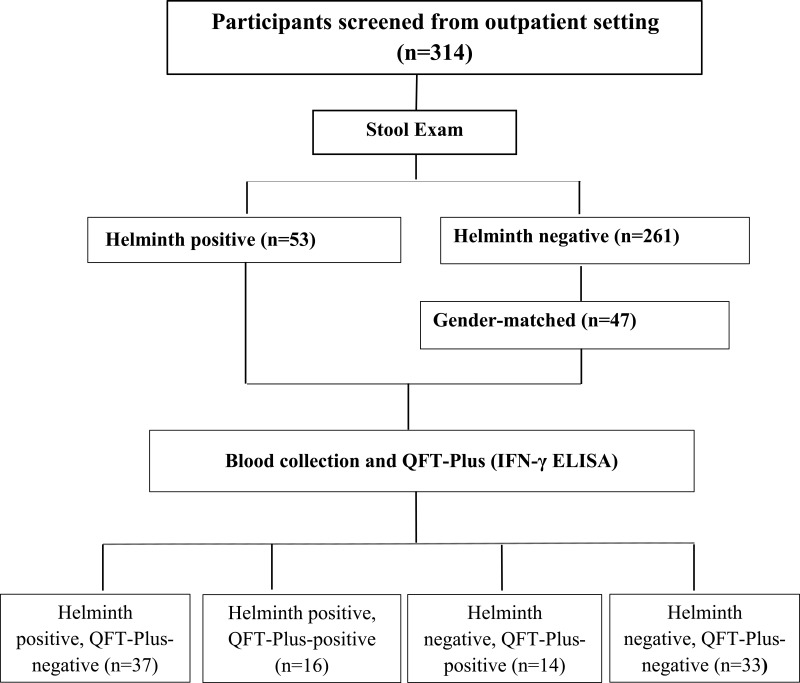
Schematic overview of participant recruitment and laboratory work (n=314): Participants were recruited from the outpatient department of Shimbit Health Center. They were screened for their eligibility criteria and those who fulfilled the criteria were selected for their stool examination and subsequent QFT-Plus test.

Participants seropositive for HIV, HBV or with evidence of clinical anemia, overt signs of acute infection, history of TB disease during the last 2 years, evidence of immunosuppressive disease conditions, on immunosuppressive treatment or pregnant women who were positive for hCG were excluded from the study. Hence, from the initial screening of eligible participants (n = 314), we identified 53 helminth-infected and 47 helminth non-infected individuals, and grouped them as cases or controls based on their helminth infection status for further analysis (Figure 1).

### Parasitological Examination

From each study participant, about 3gm of stool sample was collected with a clean screwed plastic stool cup. Within 30 minutes of stool sample collection, duplicates of slides were prepared for each of the Kato-Katz and wet mount examinations. The slides were examined by two experienced laboratory professionals independently. When there were discordant results between the two examiners, the third laboratory expert examined the slides to settle the discordant result. The wet mount preparations were examined within 30 minutes and Kato-Katz slides were examined within 30–60 minutes as per the WHO recommendation.^26^ For identification of *S. haematobium*, urine sample was also examined. The study participants were considered as helminth-positive, when at least one helminth ova and/or larva with at least one of the screening methods was detected, and the total egg count per gram of stool (EPG) was determined by the Kato-Katz slides and averaged from the two examiners.

### QuantiFERON-TB Gold Plus Test

QuantiFERON-TB Gold Plus is the latest LTBI screening test with a compartment of four tubes (Nil, TB1, TB2, and Mitogen). The Nil and Mitogens are negative and positive controls, respectively, whereas TB1 and TB2 are *M.tb* antigens. The QFT-Plus test employs a peptide cocktail simulating culture filtrate protein-10 (CFP-10) and early secretory antigenic target-6 (ESAT-6) proteins to stimulate cells in heparinized whole blood. ESAT-6 and CFP-10 are the major immunodominant antigens of *M.tb*.^27^ Both are encoded by region of difference 1 (RD1), which is present in all *M.tb* strains, but not in the vaccine strain *M. bovis* BCG.

About 5 mL of venous blood sample was collected from each study participant with lithium-heparin tube. Blood samples were transported to Amhara Public Health Institute (APHI) within three hours of blood collection. After mixing the collected blood samples, one mL of blood was transferred to each of the four QFT-Plus tubes and mixed gently ten times by inverting up and down to ensure the appropriate contact of blood with antigens or mitogen. Subsequently, the tubes were incubated at 37°C for 16 to 24 hours. After incubation, plasma was harvested following centrifugation at 3000 rpm for 15 minutes at room temperature and stored at −80°C. The stored plasma samples were then transported to the Armauer Hansen Research Institute (AHRI) with dry ice for the ELISA experiment, which was performed according to the QFT-Plus kit protocol. The optical densities (ODs) values were measured using Emax^®^ plus microplate reader, and the IFN-γ concentration in the sample was calculated based on the curve generated from the standards using QFT-Plus Analysis Software. The concentration of IFN-γ was used for the interpretation of quantitative QFT-Plus results, and qualitative results were defined based on the manufacturer’s algorithm.

### Interpretation of QFT-Plus Result

The QFT-Plus assay was interpreted as follows: The Nil control baseline IFN-γ level should be low (must be <8 IU/mL) to make sure the test interpretable, usually near 0 IU/mL. Mitogen positive control stimulates all T cells to produce IFN-γ. The Mitogen minus Nil value should be greater than 0.5 IU/mL to allow for proper test interpretation. The TB1 Antigen exposes CD4 (helper cells) to peptides from 2 proteins (CFP-10 and ESAT-6) only found in TB and sees if the immune system recognizes them and secretes IFN-γ. When the value of TB1 minus Nil and/or TB2 minus Nil was greater than 0.35 IU/mL, the result was interpreted as positive. TB2 Antigen stimulates not only CD4 cells but also CD8 (cytotoxic T cells) protein epitopes only found in TB (22). The IFN-γ concentration greater than 0.35 IU/mL with at least one of the TB1 or TB2 was considered a positive result for LTBI.

### Statistical Analysis

Data was double-entered, cleaned, and verified into the project-specific REDCap database. The data analysis was executed using SPSS version 20 statistical packages, and Graph pad prism version 6 was also used for graphical presentations. Binary logistic regression and chi-square test were employed to determine the association of QFT-Plus positivity with helminth infection and associated risk factors (age, gender, BMI, residence, occupation, and marital status). Descriptive analysis including, mean, standard deviation, frequency, and percentages were used. Odds Ratio (OR), Chi-square, *P value*, and 95% Confidence Intervals (CI) were calculated, and the result was considered statistically significant at *P value <0.05.*

## Results

### Socio-Demographic Characteristic

Initially, a total of 314 eligible participants were screened for their helminth status. Among those, 53 were infected with one or more helminths (16.87%) (Figure 1). For further QFT-Plus experiment and subsequent data analysis, we selected 100 gender-matched study participants (53 helminth-positives and 47 helminth-negatives). The mean age and mean BMI of the study participants were 28.51 (SD = 9.76) and 21.0 (SD = 2.73), respectively. Majority of the study participants were urban dwellers (95.0% (95/100)). The detailed socio-demographic and other related variables of the study participants are described in Table 1.

**Table 1:** Socio-Demographic Profile of the Study Participants, Northwest Ethiopia, 2023 (n = 100)

**Variables**	**Frequency (%)**
**Age**	
14–26	50 (50.0)
27–39	35 (35.0)
40–52	12 (12.0)
53–65	3 (3.0)
**Sex**	
Male	50 (50.0)
Female	50 (50.0)
**BMI**	
<18.5	14 (14.0)
18.5–24.9	79 (79.0)
25–29.9	5 (5.0)
≥30	2 (2.0)
**Residence**	
Rural	5 (5.0)
Urban	95 (95.0)
**Occupation**	
Gov./private employed	12 (12.0)
Self-employed	19 (19.0)
Unemployed	20 (20.0)
Farmer	2 (2.0)
Housewife	14 (14.0)
Other	33 (33.0)
**Marital status**	
Married	46 (46.0)
Single	38 (38.0)
Divorced	16 (16.0)
**Evidence or history of an allergic reaction:**	
Yes	4 (4.0)
No	96 (96.0)

**Abbreviation**: BMI, Body Mass Index.

### Helminth Infection and Associated Factors

**Figure 2:**
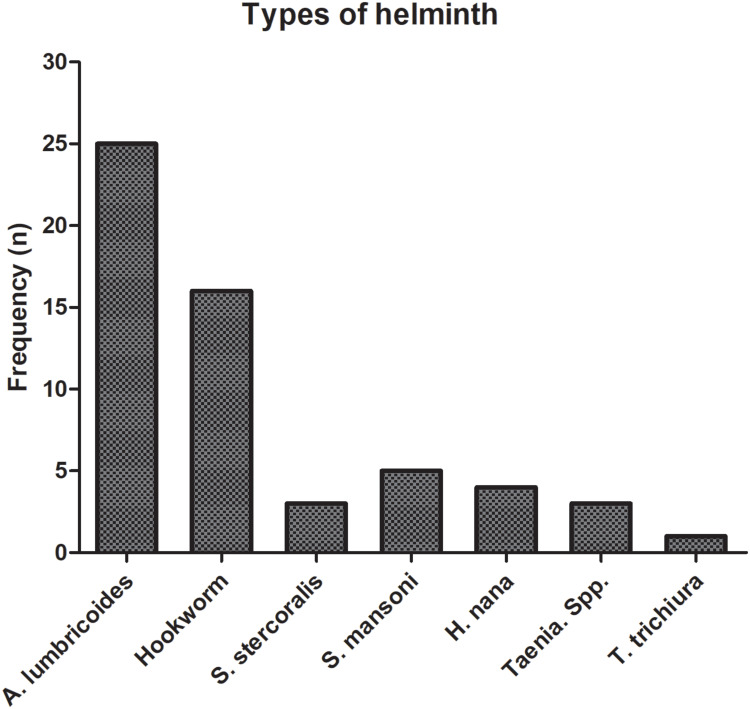
Helminth species identified (n=100): The diagnosis was performed by wet mount and Kato-Katz examination techniques and each bar represent the frequency of helminths identified by any of the stool examination method.

**Figure 3:**
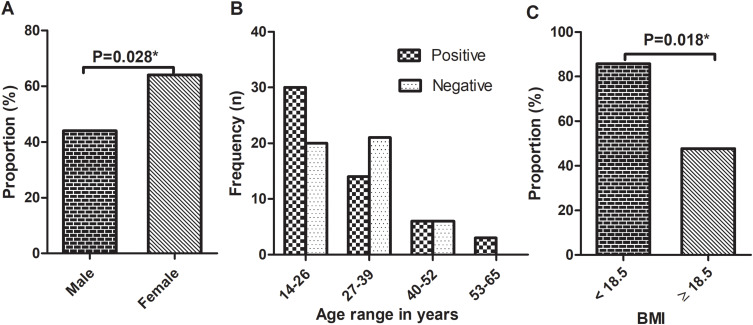
Helminth infection status (n=100) by; Gender (**A**), Age distribution (**B**), and BMI (**C**). The age range was established by class width calculation (Max-Min)/n, where “n” is the number of classes. Binary logistic regression analysis and chi-square test were applied and the *P value <0.05* was considered statistically significant. *Statistically significant.

**Abbreviations**: BMI, Body Mass Index.

Seven intestinal helminths-species were identified from 53 helminth-positive participants. Of 53, four of them had mixed infection. Specifically, *A. lumbricoides* was the most common detected, intestinal helminth (47.2% (25/53)), followed by hookworm accounting 30.2% (16/53) (Figure 2). Helminth infection was higher among female (64% (32/50)) participants than male (42% (21/50)), and the difference was statistically significant (*P = 0.028*) (Figure 3A). The proportion of helminth infection was highest among participants in the age group of 14–26 years, 56.6% (30/53) (Table 2, Figure 3B). Majority of the study participants (79.0% (79/100)) had normal BMI range (Table 1). However, among those with a BMI <18.5, 85.7% (12/14)) were infected with one or more helminths, and the difference was statistically significant (*P = 0.018*) (Figure 3C).

**Table 2:** Association of Variables to Helminths Infection Among Study Participants, Northwest Ethiopia, 2023 (n = 100)

**Variables**	**Helminth status**		**COR (95% CI)**	**AOR (95% CI)**
	**Positive N (%)**	**Negative N (%)**		
**Age**				
14–26	30 (60.0)	20 (40.0)	0.44 (0.18, 1.07)	---
27–39	14 (40.0)	21 (60.0)	0.66 (0.19, 2.36)	
40–52	6 (50.0)	6 (50.0)	---	
53–65	3 (100.0)	0 (0.0)	1	
**Sex**				
Male	21 (42.0)	29 (58.0)	1	1
Female	32 (64.0)	18 (36.0)	2.45(1.097, 5.49)*	2.32 (1.92, 5.74)*
**BMI**				
<18.5	12 (85.7)	2 (14.3)	1	---
18.5–24.9	37 (48.7)	39 (51.3)	0.16 (0.03, 075)*	
25–29.9	2 (25.0)	6 (75.0)	0.056 (0.01, 0.49)*	
≥30	2 (100.0)	0 (0.0)	---	
**Residence**				
Rural	5 (100.0)	0 (0.0)	---	---
Urban	48 (50.5)	47 (49.5)		
**Occupation:**				
Gov./private employed	0 (0.0)	12 (100.0)	---	---
Self-employed	11 (57.9)	8 (42.1)	0.44 (0.131, 1.47)	
Unemployed	6 (30.0)	14 (70.0)	0.13 (0.04, 0.47)*	
Farmer	2 (100.0)	0 (0.0)	---	
Housewife	9 (64.3)	5 (35.7)	0.57(0.15, 2.22)	
Other	25(75.8)	8 (24.2)	1	
**Marital status:**				
Married	21 (45.5)	25 (54.3)	1.21 (0.36, 4.02)	1.33(0.28, 6.22)
Single	22 (57.9)	16 (42.1)	0.61 (0.257, 1.45)	0.74 (0.25, 2.18)
Divorced	10 (62.5)	6 (37.5)	1	1

**Note**: * P-value <0.05.

**Abbreviations**: BMI, Body Mass Index; COR, Crude Odds Ratio; AOR, Adjusted Odds Ratio.

### QFT-Plus Positivity Rate and Determinant Factors

**Figure 4:**
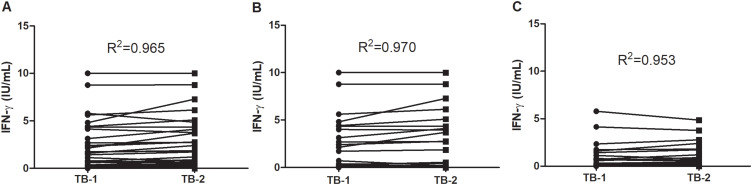
Correlation of TB1 and TB2: All participants (n=100) (**A**), Helminth positives (n=53) (**B**) and Helminth negatives (n=47) (**C**). The correlation test was analyzed with Graph pad prism version 6, and the points connected with lines fitted to indicate the deviation of points from the correlation line.

The QFT-Plus results showed IFN-γ levels above the manufacturer-recommended cut-off value of 0.35 IU/mL (in response to TB1 or TB2 stimulation) in 30 out of 100 participants. As individuals with signs and symptoms of TB disease were excluded, QFT-Plus positivity indicated LTBI in 30% of our study population. Among QFT-Plus positive participants 23.3% (7/30) had discordant results for stimulation with TB1 and TB2, of which 10% (3/30) were TB1+/TB2- and 13.3% (4/30) were TB1-/TB2+. Based on the cut-off value, the overall correlation between TB1 and TB2 was high (R^2^ = 0.965; *P < 0.001*) (Figure 4A). Moreover, the correlations between TB1 and TB2 among helminth positive (R^2^=0.970; *P < 0.001*) (Figure 4B) and helminths-negative (R^2^ = 0.953; *P < 0.001*) (Figure 4C) participants were quite similar.

A borderline range (0.20 to 0.70 IU/mL) around the cut-off level (0.35 IU/mL) was assessed, which is suggested for the earlier QFT reversion or conversion.^28^,^29^ From QFT-Plus results of the 100 participants in the present study, 8% (8/100) were borderline negative (0.20 to 0.34 IU/mL), 6% (6/100) were borderline positive (0.35 to 0.7 IU/mL), 24% (24/100) were positive (≥7.0 IU/mL), and 2% (2/100) had indeterminate result.

**Figure 5:**
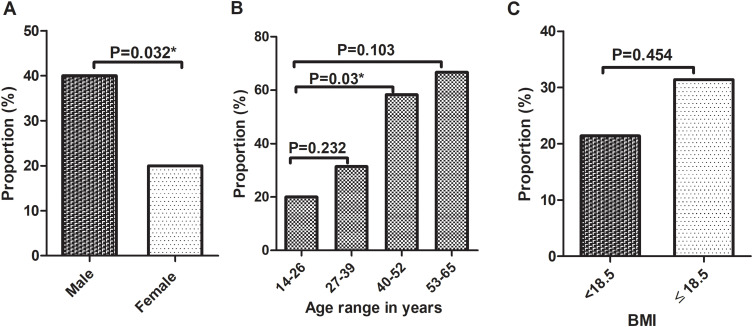
QFT-Plus positivity distribution of study participants (n=100) by; Gender (**A**), Age (**B**), and BMI (**C**). The bar graphs showed the proportions of QFT-Plus positivity. The *P value <0.05* was considered statistically significant. *Statistically significant.

**Abbreviations**: BMI, Body Mass Index.

The proportion of QFT positivity was higher among male participants (40% (20/50)) than female participants (20% (10/50)), and the difference was statistically significant (OR = 2.93; 95% CI: 1.07, 8.00; *P = 0.032*) (Table 3, Figure 5A). QFT-Plus positivity was also significantly higher in the 40–52 age group compared to the 14–26 age group (OR = 5.32, 95% CI: 1.34, 21.12; *P = 0.018*). Additionally, there was an increasing trend in QFT-plus positivity with age (Figure 5B). Conversely, QFT positivity was lower among participants with a BMI <18.5, (21.4% (3/14)) compared to those with a BMI ≥18.5, (31.4% (27/86)), though, the difference was not statistically significant (Figure 5C).

**Table 3:** Association of Determinant Factors with QFT Positivity Among Study Participants, Northwest Ethiopia, 2023 (n = 100)

**Variables**	**QFT result**		**COR (95% CI)**	**AOR (95% CI)**
	**Positive N (%)**	**Negative N (%)**		
**Age**				
14–26	10 (20.0)	40 (80.0)	1	1
27–39	11 (31.4)	24 (68.6)	1.83 (0.68, 4.98)	1.89 (0.59, 6.00)
40–52	7 (58.3)	5 (41.7)	5.6 (1.46, 21.40)*	6.37 (1.41, 28.67)*
53–65	2 (66.7)	1 (33.3)	8.0 (0.65, 97.31)	9.30 (0.64, 134.18)
**Sex**				
Male	20 (40.0)	30 (60.0)	2.67 (1.09, 6.52)*	2.93 (1.07, 8.00)*
Female	10 (20.0)	40 (80.0)	1	1
**BMI**				
<18.5	3 (21.4)	11(78.6)	1	---
18.5–24.9	25 (31.6)	54 (68.4)	1.79 (0.46, 7.02)	
25–29.9	2 (40.0)	3 (60.0)	1.22 (0.15, 9.46)	
≥30	0 (0.0)	2 (100.0)	---	
**Residence**				
Rural	1 (20.0)	4(80.0)	1	1
Urban	29 (30.5)	66(69.5)	1.76 (0.19, 16.41)	2.67 (0.18, 38.86)
**Occupation:**				
Gov./private employed	3 (25.0)	9 (75.0)	0.76 (0.17, 3.44)	---
Self-employed	7(36.8)	12 (63.2)	1.34 (0.40, 4.41)	
Unemployed	6 (30.0)	14 (70.0)	0.98 (0.29, 3.30)	
Farmer	1 (50.0)	1 (50.0)	2.3 (0.13, 40.54)	
Housewife	3 (21.4)	11(78.6)	0.62 (0.14, 2.74)	
Other	10 (30.3)	23(69.7)	1	
**Marital status:**				
Married	19 (41.3)	27 (58.7)	2.11 (0.59, 7.55)	---
Single	7 (18.4)	31 (81.6)	0.67 (0.16, 2.74)	
Divorced	4 (25.0)	12 (75.0)	1	
**Helminth**				
Positive	16 (30.2)	37 (69.8)	1	1
Negative	14 (29.8)	33 (70.2)	1.09 (0.43, 2.40)	1.45 (0.51, 4.09)

**Note**: * P-value <0.05.

**Abbreviations**: BMI, Body Mass Index; COR, Crude Odds Ratio; AOR, Adjusted Odds Ratio.

### Effect of Helminth Species and Parasite Load on QFT-Plus Test Outcome

**Figure 6:**
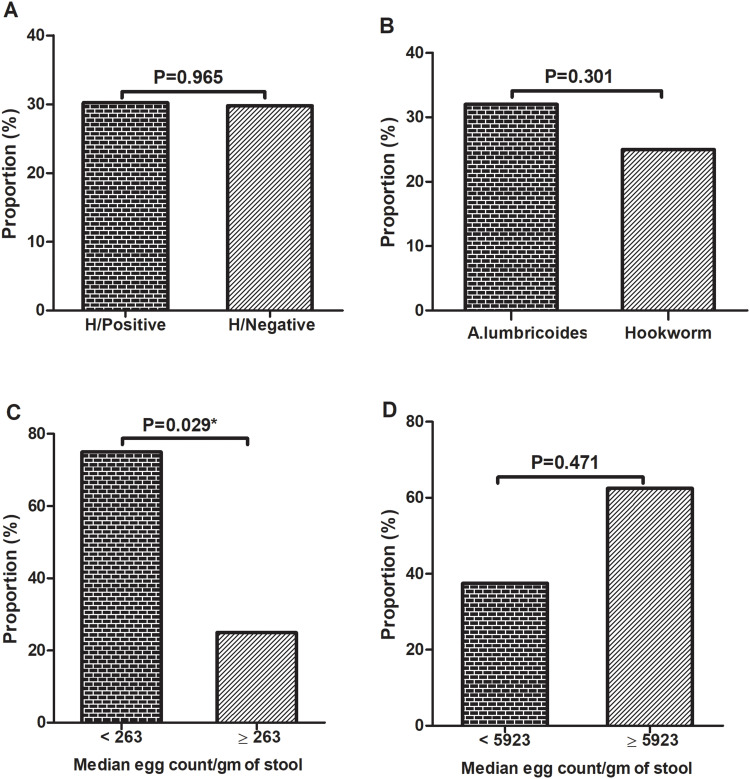
QFT positivity and helminth infection: QFT positivity by helminth status (n=100) (**A**), QFT+ helminth species; hookworm (n=16) (25%, (4/16)), *A. lumbricoides* (n=25) (32% (8/25)) (**B**), QFT+ hookworm (n=4) (<MEC Vs ≥MEC:75% (3/4) Vs 25% (1/4)) (**C**), and QFT+ *A. lumbricoides* (n=8) (<MEC Vs ≥MEC:37.5% (3/8) Vs 62.5% (5/8)) (**D**). The cut-off values for the egg count of hookworm and *A. lumbricoides* were established based on the median egg count of each parasite. The *P value <0.05* was considered statistically significant. *Statistically significant.

**Abbreviations**: MEC, Median Egg Count.

The proportions of QFT-Plus positivity among the helminth positive and negative participants were 30.2% (16/53) and 29.8% (14/47), respectively (*P = 0.965*) (Table 3, Figure 6A). Since soil-transmitted helminths (STH) are most common in Ethiopia, we tried to assess the QFT-Plus positivity rate among STH positive and negative participants. From the 53 helminth positive individuals, 44 of them were infected with STH. Of the 44 STH infected individuals the proportion of QFT-Plus positivity rate was 29.5% (13/44), which was also comparable to helminth-negative participants (29.8% (14/47). Considering the two predominantly identified helminth species, the proportion of QFT-Plus positivity rate was lower in participants infected with hookworm compared to *A. lumbricoides* (Figure 6B). However, the difference was not statistically significant.

Following WHO classification, we categorized hookworm infected participants into light (<1, 999 EPG), moderate (2000–3999 EPG) and heavy (≥4,000 EPG) infection groups.^30^ However, all but two participants (one with moderate and one with heavy infection) were classified under the light infection category. Similarly, for *A. lumbricoides,* we identified 10 participants with light infection (1–4999 EPG), 14 with moderate infection (5000–49,999 EPG), and one with heavy infection (≥50,000 EPG). Due to the small number of participants in these categories, we used median egg count for statistical analysis. Using the median egg count, the QFT-Plus positivity rate was significantly lower in participants with hookworm egg counts above the median (*P = 0.029*) (Figure 6C). However, this trend was not observed in participants with *A. lumbricoides infection* (*P = 0.471*) (Figure 6D).

## Discussion

Helminth and *M. tb* infections are common in third-world countries, including Ethiopia.^8^,^31^,^32^ The outcome of the co-infection mainly depends on different immunological responses including cytokine/chemokine dynamics.^33^ It is well established that intestinal helminth infection leads to the expansion of a Th2-dominated immune response and Th3 immune-regulatory response^33–35^ that can mitigate the Th1 and Th17 type of immunity,^36^ which is essential for defense against TB, and cytokine-based IGRA tests for the diagnosis of LTBI.

In the present study, the overall prevalence of helminth infection was 16.87%, in which *A. lumbricoides* and hookworm were the two predominantly identified helminth species. Likewise, the two most prevalent species were reported by a recently published systematic review^5^ and individual studies conducted in Amhara and Oromia regions, Ethiopia.^37^,^38^ Though *S. mansoni* is highly prevalent in Bahir Dar and surroundings^39^,^40^ only a few participants were positive in our study (Figure 2). The possible reason might be that the majority of studies conducted among school-age children (6 to 12 years). Children in this age group are most vulnerable to *S. mansoni* infection due to certain play habits such as swimming in infested water bodies.^38^,^41^ However, our study was conducted largely on adult participants, mostly from an urban community.

Low BMI was significantly associated with helminth infections (*P = 0.018*) (Figure 3C). A similar finding has been reported by Alemu et al.^8^ Helminths are parasitic worms that depend on a living host for food and shelter while causing poor nutrient absorption that compromises the nourishment, and subsequently an immune response of the host.^42^ Studies revealed reduced numbers of T cells and their function among malnourished individuals.^43^,^44^ Thus, parasite infection could be considered as one of the predisposing factors for low BMI, resulting in reduced IFN-γ production and the progression of LTBI to active TB.

In the current study, the result of QFT-Plus indicated that about one-third (30.0%) of the study participants were LTB-infected. This finding is lower than the results of other studies conducted in Ethiopia.^45^,^46^ The possible reason could be a different age distribution of study participants; for example in Tilahun et al study, >60% were older than 30 years, whereas in our case 50% were younger than 27 years (Table 1). Nevertheless, our result is similar to the general population as noted by the WHO, which estimates around 30%.^47^ On the other hand, our finding is higher compared to the study conducted among Nepal migrants in the United Kingdom (UK) (15.8%).^48^ The lower prevalence of LTBI among Nepal migrants might be due to population difference and the lower age group of study participants (median age was 19.3 years (IQR 18.7–20.0 years)). From the total QFT-Plus result, 14% (14/100) were borderline (0.20 to 0.7 IU/mL) and the proportion of the borderline result was higher among helminth positive participants compared to negatives (16.9% vs 10.6%), but the difference was not significant (*P = 0.195*). The borderline results, which are considered a zone of uncertainty, could have a high conversion or reversion rate.^49–51^

In this study, there was a gender-based difference in QFT-Plus positivity where being male was 2.93 times more likely to be QFT-Plus positive compared to female participants (*P = 0.032*). This finding is in line with the studies done in Ethiopia and Latin America,^46^,^52^ while other studies showed no gender-based association.^53^,^54^ The possible reason might be; first, culturally males are more exposed to outdoor social activities in the study area, which may surge the probably of *M. tb* exposure; secondly, in the current study the number of helminth positivity, particularly hookworm (56.3% (9/16)) infection, was higher among females than males that could induce a Th2 response, which led to an inverse association with QFT positivity in our study (Figure 6C).

Though the difference was not statistically significant, QFT positivity was lower among participants with a BMI <18.5 compared to those with a BMI ≥18.5 (Figure 5C). In the current study, helminth positivity was higher among participants with a BMI <18.5, while QFT positivity was lower (Figure 3C, Figure 5C). This might compromise the *M.tb*-specific immune response (primarily IFN-γ production) of the host.

QFT positivity was significantly higher among the age group 40–52 years compared to the age group 14–26 years (*P = 0.03*) and marginally showed increasing trends with the age group of study participants, which is in keeping with other data.^46^,^52^,^54^ One possible reason might be the higher probability of cumulative exposure to *M.tb* with increasing age. On the other hand, helminth infection showed a decreasing trend in the higher age group, while QFT positivity inversely increased in the current study (Figure 5B). Thus, helminth infection, specifically hookworm infection, could be responsible for the lower QFT-Plus positivity. Even though aging is another possible factor for immunodeficiency, this is usually common in elderly people (≥65 years).^55^ In our study, the age range was 14–65 years, and the majorities (85.0%) of participants were below the age group of 40 years. Hence, the role of old age-related immunodeficiency in affecting QFT-Plus positivity could be less likely.

In our study, the QFT-Plus positivity rate was comparable among helminth positive and negative participants. The majority of participants resided in urban areas, which along with other idiopathic host-related factors may have contributed to the observed low association between helminth infections and QFT-Plus test performance. We used egg counts in stool samples as a surrogate measure of the helminth parasite density determines the infection’s intensity. While the relationship between parasite density and QFT positivity is not well established, some studies suggest that helminth infections are associated with increased levels of regulatory T cells and IL-10, which may suppress the Th1 response.

Although the overall helminth infection did not show an association with QFT-Plus positivity in this study, we sought to determine whether parasite density could impact QFT results for the two predominant parasites identified. We found that QFT positivity rates were significantly lower in hookworm-infected individuals with egg counts above the median value (*P = 0.029*) (Figure 6C). A study from Uganda showed that HIV-positive participants with hookworm infection had lower average CD4^+^ cell counts than participants infected with other helminths.^56^ Another study detected a lower frequency of activated CD4^+^ and CD8^+^ T cells among TB-hookworm co-infected group as compared to healthy controls and the non-co-infected pTB patients.^57^ Moreover, the study done by George et al demonstrated a significant inhibitory effect of coincident hookworm infection on protective cell-mediated immune cells (Th1 and Th17) response in LTBI.^58^ In contrast, QFT positivity was slightly increased while the parasite load of *A. lumbricoides* increased, which is an inverse finding compared to hookworm, but the difference was not statistically significant (Figure 6D). *A. lumbricoides* is known to be a benign infection that may not have strong effect on IFN-γ production.^25^ A study conducted in Venezuela reported that *A. lumbricoides*-modulated Th2 and IL-10 levels can increase the result of a positive TST after *M. tb* exposure.^36^ Another study in Brazil showed that *A. lumbricoides* infection had no impact on Th1, Th2 and Th17 responses.^59^ Further studies about immune modulation of helminth species and parasite intensity with large sample sizes are suggested.

Though this study delivers valuable information about helminth infection and the new IGRA test called QFT-Plus, it has some limitations. The first limitation is the limited sample size, which may affect the statistical modeling and analysis. Moreover, we did not use molecular tests, known for improved sensitivity for helminth diagnosis, which may affect the accurate categorization of the study participants as it is not the gold standard.

## Conclusion and Recommendation

There was no difference in QFT-Plus test positivity between helminths positive and negative participants. However, the QFT Plus positivity rate was significantly lower when a parasitic load of hookworm became higher than the median egg count. Hence, hookworm infection could have a significant effect on the performance of IGRA tests compared to other helminth species. The lower QFT-Plus positivity among female participants might be due to the higher helminth infection in females than in males. This may be due to the Th2 bias that affects the performance of the QFT-Plus test. More studies with larger sample sizes and sensitive molecular diagnostic methods for helminth infection are suggested to clarify the species-specific effect of helminths on the immune response of the host and the performance of the QFT-Plus test.

## Data Availability

All data pertaining to this study are contained and presented in this document.

## Abbreviations

AHRI, Armauer Hansen Research Institute; APHI, Amhara Public Health Institute; BMI, Body Mass Index; CDC, Centers for Disease Control and Prevention; CFP-10, Culture Filtrate Protein-10; ELISA, Enzyme Linked Immunosorbent Assay; ESAT-6, Early Secretory Antigenic Target-6; hCG, Human Chorionic Gonadotropin; IDSA, Infectious Disease Society of America; IFN-γ, Interferon Gamma; IGRA, Interferon Gamma Releasing Assay; IL, Interleukin: LTBI, Latent TB Infection; *M.tb, Mycobacterium tuberculosis*; OD, Optical Densities; PPD, Purified Protein Derivative; QFT-Plus, QuantiFERON-TB Gold Plus; SPSS, Statistical Package for the Social Sciences; STH, Soil Transmitted Helminth; TB, Tuberculosis; TST, Tuberculin Skin Test; WHO, World Health Organization.
